# Loss of capture of conduction system pacemaker caused by fibrosis surrounding the lead: a case report

**DOI:** 10.1186/s12872-023-03656-3

**Published:** 2023-12-19

**Authors:** Luuk H.G.A. Hopman, Kyle P. Beunder, Sonia Borodzicz-Jazdzyk, Marco J.W. Götte, Vokko P. van Halm

**Affiliations:** 1 Department of Cardiology, Amsterdam UMC, Amsterdam, The Netherlands; 2 1st Department of Cardiology, Medical University of Warsaw, Warsaw, Poland

**Keywords:** Conduction system pacing, Cardiac MRI, Fibrosis, AV-block

## Abstract

**Background:**

Conduction system pacing (CSP) is a novel technique that involves pacing the His-Purkinje system instead of the traditional right ventricular (RV) apex. This technique aims to avoid the adverse effects of RV apical pacing, which can lead to ventricular dyssynchrony and heart failure over time. CSP is gaining popularity but its long-term efficacy and challenges remain uncertain. This report discusses a case where CSP was initially successful but faced complications due to an increasing pacing threshold.

**Case presentation:**

A 65-year-old female with total atrioventricular block was referred for brady-pacing. Due to the potential for chronic RV pacing, CSP was chosen. The CSP implantation involved subcutaneous device placement, with a CSP lead in the left bundle branch area (LBBA) and an RV backup lead. A year after successful implantation, the LBBA pacing threshold progressively increased. Subsequent efforts to correct it led to anodal capture and battery depletion. Cardiac magnetic resonance imaging (CMR) revealed mid-septal fibrosis at the area of LBBA lead placement and suggested cardiac sarcoidosis as a possible cause.

**Conclusion:**

CSP is a promising technique for treating bradyarrhythmias, but this case underscores the need for vigilance in monitoring pacing thresholds. Increasing thresholds can render CSP ineffective, necessitating alternative pacing methods. The CMR findings of mid-septal fibrosis and the potential diagnosis of cardiac sarcoidosis emphasize the importance of pre-implantation assessment, as CSP may be compromised by underlying structural abnormalities. This report highlights the complexities of pacing strategy selection and the significance of comprehensive evaluation before adopting CSP.

## Background

For many years, right ventricular (RV) pacing has been the standard strategy for treating symptomatic bradyarrhythmias [1]. However, in recent years, conduction system pacing (CSP) has become increasingly popular and is now widely used in leading cardiac centers across Europe [2]. CSP is an innovative pacing technique that involves implanting a pacing lead directly into the His-Purkinje system instead of the traditional RV apex [3]. It has been shown that RV apical pacing gives rise to electrical and mechanical ventricular dyssynchrony, which can impair cardiac function and may cause heart failure (HF) over time [4]. This has been substantiated by numerous studies, reporting a higher incidence of HF hospitalization and atrial fibrillation in patients who receive a high burden of RV pacing [5, 6]. In contrast, CSP more closely mimics the natural electrical activation of the heart and this may better preserve cardiac function, especially in patients with a preserved left ventricular (LV) ejection fraction (LVEF) who are expected to have more than 20% ventricular pacing [7].

Despite studies demonstrating the safety and feasibility of CSP, concerns about this innovative technique still exist and long-term follow-up data alleviating these concerns is limited [2, 8, 9]. In addition, CSP and especially His-pacing appears to be a more challenging implantation procedure associated with higher conduction system capture thresholds at implant and sometimes even loss of conduction system capture during follow-up, which may compromise the effectiveness of the pacing therapy [10].

We recently encountered a case of CSP in which the pacing threshold gradually increased over the year after implantation, eventually rendering the CSP lead placed in the left bundle branch area (LBBA) unusable. Therefore, the RV backup lead had to be used for pacing, increasing the risk for HF in the long term.

In this case report, we highlight the need for (close) monitoring of pacing thresholds in CSP patients, as well as the importance of performing a cardiac magnetic resonance imaging (CMR) scan for unraveling the underlying cause of atrioventricular (AV)-block to consider placing a back-up lead in patients who are pacemaker-dependent.

## Case presentation

The present case involves a 65-year-old female with a medical history of hypertension and type 2 diabetes mellitus. In 2020, her ECG showed complete left bundle branch block (LBBB), and in January 2021 the patient experienced dizziness with no apparent cardiac cause. In October 2021, the patient was seen in the outpatient clinic because of dizziness and shortness of breath. ECG and Holter monitoring revealed a 2:1 AV-block, high-degree AV-block, and complete AV-block. A trans-thoracic echo showed normal cardiac function, except for the abnormal septal movement typically seen in LBBB. Subsequently, the patient was referred to our center for brady-pacing and due to the prospect of chronic RV pacing, she was accepted for CSP.

Upon admission for CSP implantation in October 2021, the patient had complete AV-block with a ventricular escape rhythm of 33 beats per minute. The CSP implantation was successfully performed under local anesthesia with the device placement location being subcutaneous and left-sided. The right atrial lead was successfully positioned within the right atrial appendage. Consistent with the 2021 ESC pacing and CRT guidelines (Class IIa recommendation), the operator proceeded to insert an RV backup lead at the RV apex in the context of His-pacing.

**Fig. 1:**
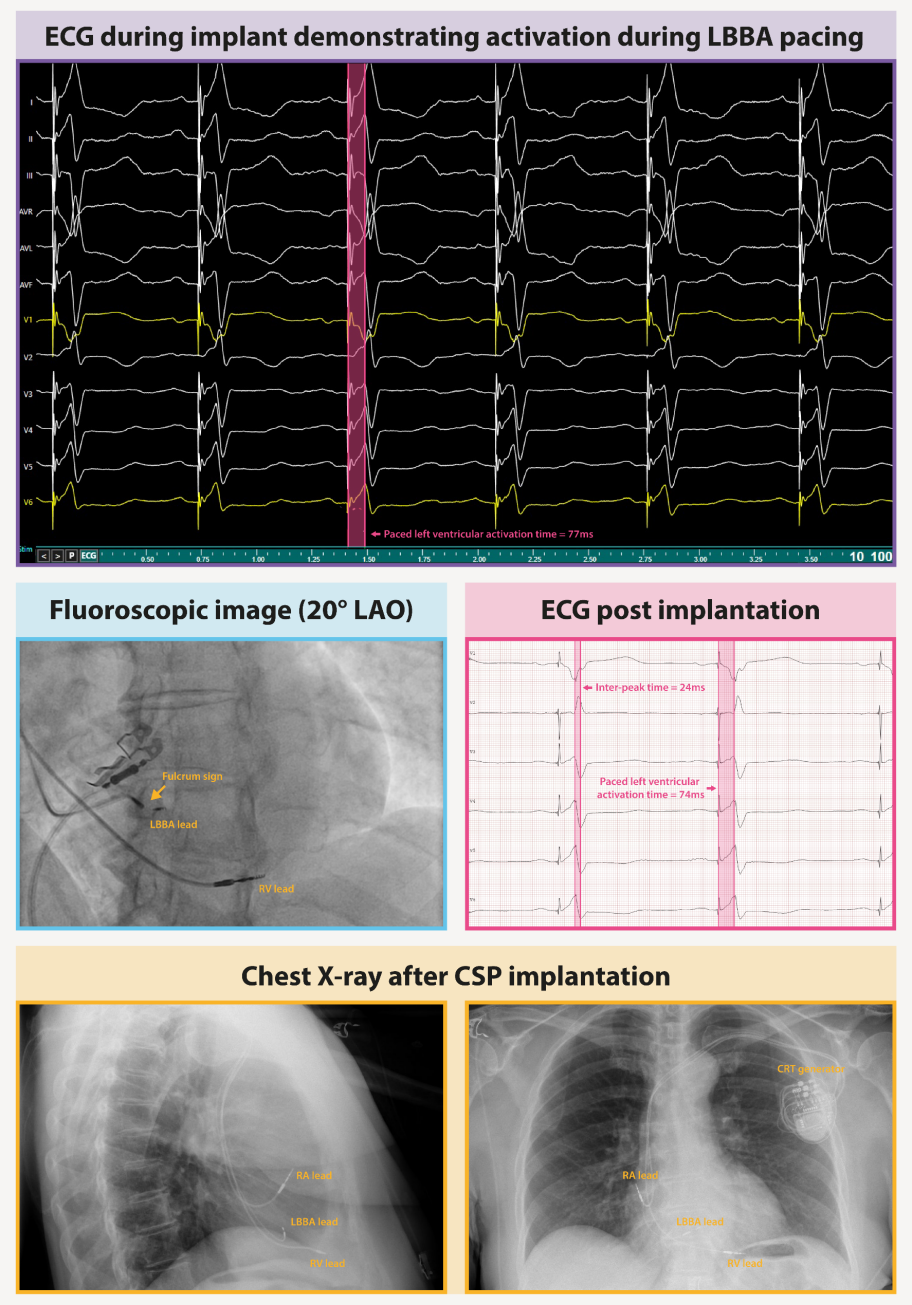
Conduction system pacing implantation. LBB pacing lead implantation in a patient with AV-block, with electrograms during LBB pacing. The paced LV activation time is 77ms. In the middle left panel, a fluoroscopy image reveals the fulcrum sign, illustrating the pivotal point where the lead body intersects with the RV septum. Chest X-rays are presented below, in which the leads and generator are visible

Thereafter, a ventriculogram was performed to visualize the His-region for CSP lead placement (Medtronic SelectSecure Model 3830 lumen less lead, Medtronic, Inc., Minneapolis, MN). His capture could be achieved at several sites, but pacing at high output only resulted in partial correction of the LBBB. After three unsuccessful attempts to correct the LBBB completely at the His, the decision was made to proceed with conversion to the LBBA. The CSP lead was then placed in the ventricular septum until sufficient (non-selective) LBBA pacing was achieved, with satisfying sense and threshold values. During pacing, specific observations were made; a modest R’ in V1 and a stimulus to LV activation time of less than 80ms. The inter-peak time measured was 24ms (interval between peak R-wave in V6 and R’ in V1) (Fig. 1). Achievement of more selective LV capture was hindered by the challenge of reaching deeper into the septum. Following lead implantation, a fluoroscopic image revealed the fulcrum sign, demonstrating the placement of the CSP lead tip within the RV septal wall (Fig. 1). Finally, a cardiac resynchronization therapy pulse generator (Medtronic Solara CRT-P, Medtronic, Inc., Minneapolis, MN) with the CSP lead connected to the LV port was implanted subcutaneously. The LBBA pacing threshold at implant was 0.5 V and the pacing settings were eventually programmed to 2.5 V/0.4ms for the LBBA lead.

The day after the procedure, a chest X-ray confirmed the lead position and a threshold test was performed to ensure stable pacing values (Fig. 1 lower panel). Two months after implant, during the routine device checkup, it was discovered that the LBBA pacing threshold had increased slightly to 1.25 V.

**Fig. 2:**
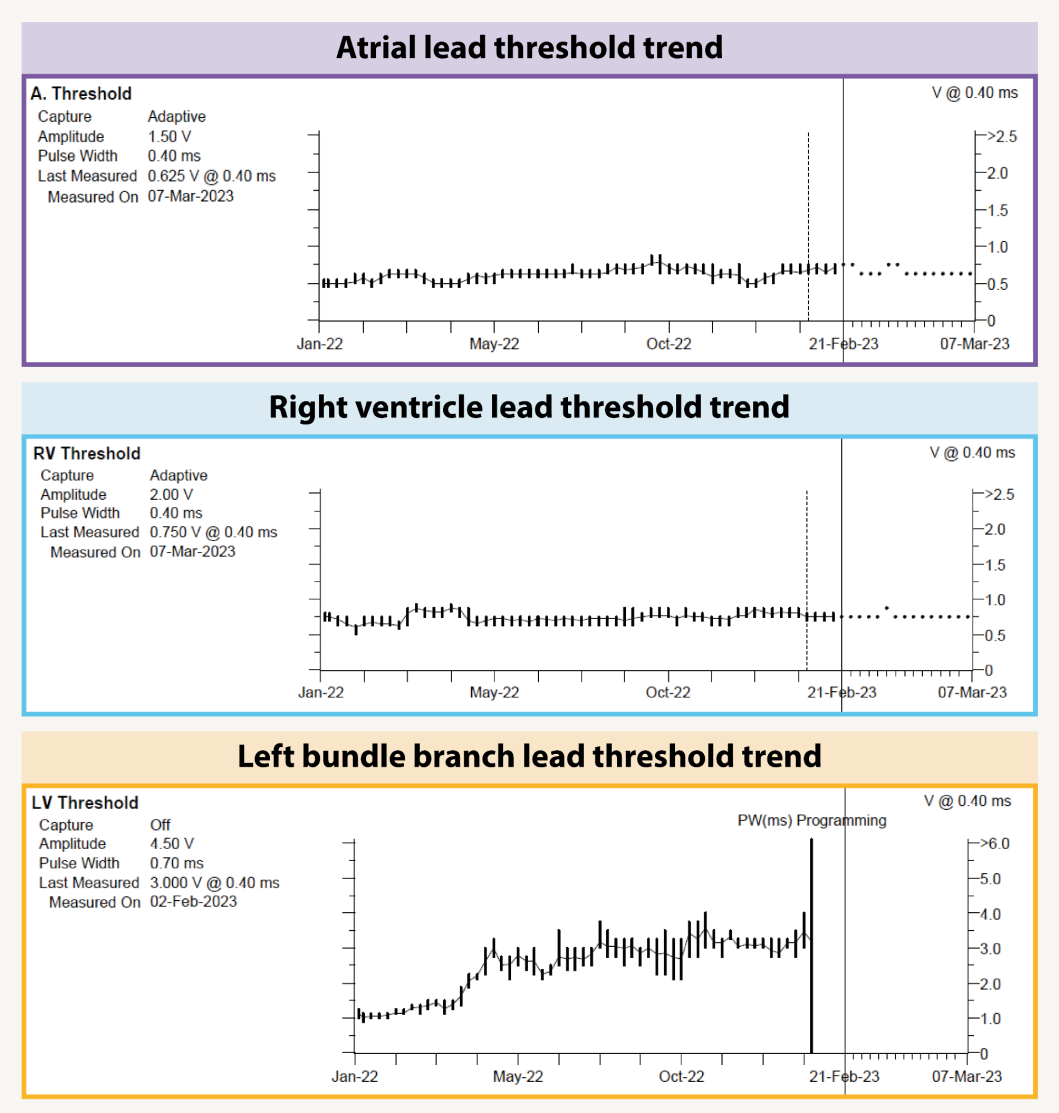
Pacing threshold trends. Threshold trends over time for the atrial lead, right ventricle lead and left bundle branch lead

During the routine device follow-up at one year, it was observed that the LBBA pacing threshold had risen to 3 V, which was above the pacing output of 2.5 V. Consequently, the patient was receiving RV only pacing from the backup lead (Fig. 2). Attempts to increase the output led to pocket stimulation, prompting a change in the pacing configuration to LVtip – RVring at 4 V/0.7ms. Subsequent follow-up two months later revealed an even higher LBBA pacing threshold. At increased pacing output, anodal capture at the lead ring was observed, and battery life expectancy decreased drastically. As a result, RV pacing was established as the default mode, and LBBA pacing was deactivated.

**Fig. 3:**
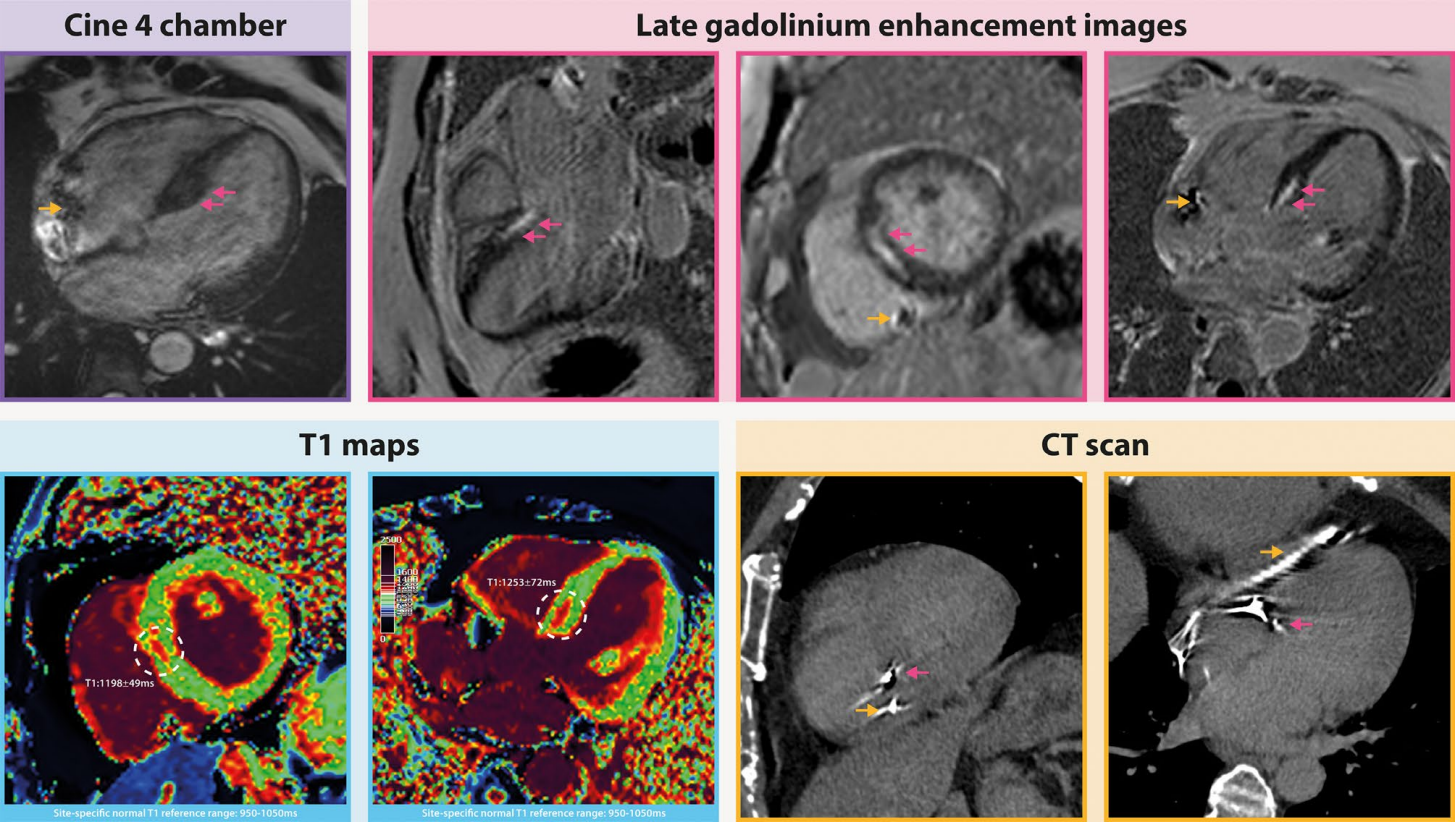
Cardiovascular MRI scan. The 4-chamber cine image showed proximal septal thickening (purple arrows) and the artifacts of the leads (yellow arrows). Late gadolinium enhancement images displayed a patchy non-ischemic mid-myocardial high signal intensity in the same proximal septum (purple arrows). T1 mapping demonstrated focal high T1 values in the proximal septum of 1263 ± 51ms (site-specific normal myocardial T1 values: 950–1050ms). On the CT scan, the lead artifacts are distinctly discernible (purple arrow is the LBBA lead, yellow arrow is the RV lead), providing a clear indication of the point at which the lead was inserted into the right ventricular septum. The insertion site aligns with the corresponding late gadolinium enhancement images within the same image plane. Notably, the late gadolinium enhancement exhibits a broader extent than the lead insertion site, encompassing both the infero-antero septal and basal-to-mid septal directions

Subsequently, a CMR scan (1.5T scanner, Sola, Siemens Healthineers, Erlangen, Germany) was performed to investigate the possible cause of the increasing LBBA pacing threshold. To ensure safety during the procedure, the device was programmed in the MR safe mode. The following imaging techniques were applied: cine imaging, T1 and T2 mapping, and wideband late gadolinium enhancement (LGE) imaging (to reduce cardiac implantable electronic device–induced artefacts) as presented in Fig. 3. The cine imaging showed a normal LVEF of 58% and normal cardiac dimensions. Proximal LV septum thickening (14 mm) was present with hypokinesia in the same area. T1 mapping demonstrated focal high T1 values in the proximal LV septum of 1263 ± 51ms (site-specific normal myocardial T1 values: 950–1050ms) indicative of myocardial fibrosis, edema or infiltration. On T2 maps, a slightly increased T2 value was present in the same area (54.6 ± 2.7ms) as compared to the site-specific normal myocardial T2 values (42–50ms), compatible with focal edema/inflammation. LGE images revealed non-ischemic patchy mid-myocardial high signal intensity in the same proximal LV septum, suggestive of focal fibrosis. Fibrosis was not limited to the insertion point of the CSP lead tip; instead, it extended in both the basal-apical and inferoseptal-inferolateral directions. The combination of findings, including basal septal thickening with mid-myocardial focal LGE and evidence of inflammation with concomitant diffuse myocardial edema is suggestive of cardiac sarcoidosis. Therefore, further lab tests and a PET-CT scan were performed. Lab results showed an increased serum angiotensin-converting enzyme (ACE), and normal soluble Interleukin2-receptor antagonist levels. PET-CT findings did not support the diagnosis of cardiac sarcoidosis (no lymphadenopathy or avid lymph nodes within the imaged region) or the presence of active myocarditis. Alternative causes for basal septal thickening with mid-wall focal LGE could be an infiltrative cardiomyopathy, systemic disease, or a hypertrophic cardiomyopathy.

## Discussion and conclusions

In recent years, CSP has become one of the most promising advancements in pacing therapy and the technique is rapidly gaining adoption in global clinical practice. However, as described in our case, septal fibrosis can significantly impede CSP therapy, necessitating RV backup lead pacing.

Our initial hypothesis proposed that the friction generated by the LBBA lead on the contracting myocardium could potentially induce fibrosis development around the lead tip. It was reasonable to speculate that the wear and tear caused by the lead on the surrounding myocardium might lead to localized damage, potentially resulting in an increase in LBBA pacing threshold. However, the results of the CMR scan conducted post CSP implantation unveiled a rather substantial area of patchy basal mid-septal fibrosis at the placement site of the lead. Notably, the fibrotic region extended beyond the confines of the lead insertion site, as evident on the LGE images, surpassing the lead’s visibility on the CT scan. This observation suggests an alternative pathological process at play, distinct from a direct impact due to lead positioning within the septum. The observed mid-septal fibrosis may have been pre-existing, and its advancement in the lead-up to and following device placement could be indicative of a progressive AV conduction disorder. This is supported by the patient’s initial diagnosis of LBBB in 2020, subsequent development of complete AV-block by the end of 2021, and challenges the operator faced to obtain selective LBB capture. Performing the CMR scan provided crucial insights into the patient’s underlying conduction disease. Despite the fact that the CRT pulse generator and three leads could potentially disrupt image quality, the CMR scan yielded high-quality results, including parametric mapping images. Employing a wideband LGE sequence significantly minimized device-related artifacts, resulting in the acquisition of high-quality images. This enhancement played a pivotal role in facilitating precise clinical interpretation.

Although the presence of AV block and the results of the CMR scan initially suggested cardiac sarcoidosis, further diagnostic testing did not provide conclusive evidence to support this diagnosis. It is possible that there was active cardiac sarcoidosis at some point, as indicated by the CMR findings and elevated serum ACE levels. At the time of serum biomarker measurement one month after the CMR scan however, the cardiac sarcoidosis may have already been subsiding. Moreover, when the PET-CT scan was performed 10 weeks after the CMR scan, the patient may have recovered without additional therapy. Alternatively, a previous episode of myocarditis, other infiltrative disease, or an underlying systemic disease could potentially account for the observed septal fibrosis. For the time being, we will maintain close monitoring over the patient to assess the impact of chronic RV pacing. If there is a decline in LV function accompanied by clinical symptoms indicative of pacemaker-induced cardiomyopathy, we will consider recommending LV lead placement to facilitate cardiac resynchronization therapy.

The presented case study has several noteworthy limitations that warrant acknowledgment. Firstly, it is essential to recognize that the presented case involves a patient with LBBA pacing, and true LBB capture was not achieved. Additionally, the absence of a cine image of the sheath venogram immediately post-lead implantation hampers our ability to assess the depth of lead insertion accurately. Furthermore, the omission of extensive pre-procedural imaging, such as CMR, introduces a gap in our comprehension of the factors contributing to the loss of LBBA pacing. Lastly, it is essential to underscore that not all medical centers possess the capability or expertise to conduct both pre- and post-implantation CMR scans of high quality for these patients, particularly in regions or among patient classes facing economic constraints.

In conclusion, while CSP represents a significant advancement in pacing therapy, it is not a panacea for all cases of AV-block. The presence of septal fibrosis, which can impair CSP therapy, emphasizes the importance of considering CMR before choosing the optimal pacing strategy and guidance of CSP lead placement in selected patients, provided that resources permit its inclusion in the diagnostic process.

## Data Availability

The datasets used and/or analyzed during the current study are available from the corresponding author upon reasonable request.

## References

[1] Brignole M, Auricchio A, Baron-Esquivias G (2013). 2013 ESC guidelines on cardiac pacing and cardiac resynchronization therapy: the Task Force on cardiac pacing and resynchronization therapy of the European Society of Cardiology (ESC). Developed in collaboration with the European Heart Rhythm Association (EHRA). Eur Heart J.

[2] Jastrzebski M, Kielbasa G, Cano O (2022). Left bundle branch area pacing outcomes: the multicentre European MELOS study. Eur Heart J.

[3] Burri H, Jastrzebski M, Cano O (2023). EHRA clinical consensus statement on conduction system pacing implantation: executive summary. Endorsed by the Asia-Pacific Heart Rhythm Society (APHRS), Canadian Heart Rhythm Society (CHRS) and latin-american Heart Rhythm Society (LAHRS). Europace.

[4] Sweeney MO, Prinzen FW (2006). A new paradigm for physiologic ventricular pacing. J Am Coll Cardiol.

[5] Kiehl EL, Makki T, Kumar R (2016). Incidence and predictors of right ventricular pacing-induced cardiomyopathy in patients with complete atrioventricular block and preserved left ventricular systolic function. Heart Rhythm.

[6] Sharma PS, Patel NR, Ravi V (2022). Clinical outcomes of left bundle branch area pacing compared to right ventricular pacing: results from the Geisinger-Rush Conduction System Pacing Registry. Heart Rhythm.

[7] Glikson M, Nielsen JC, Kronborg MB (2021). 2021 ESC guidelines on cardiac pacing and cardiac resynchronization therapy: developed by the Task Force on cardiac pacing and cardiac resynchronization therapy of the European Society of Cardiology (ESC) with the special contribution of the European Heart Rhythm Association (EHRA). Eur Heart J.

[8] Mafi-Rad M, Luermans JG, Blaauw Y (2016). Feasibility and acute hemodynamic effect of left ventricular septal pacing by Transvenous Approach through the interventricular septum. Circ Arrhythm Electrophysiol.

[9] Padala SK, Ellenbogen KA (2020). Left bundle branch pacing is the best approach to physiological pacing. Heart Rhythm O2.

[10] Keene D, Anselme F, Burri H et al. Conduction system pacing, a European survey: insights from clinical practice. *Europace* 2023. 10.1093/europace/euad019 PMC10227660 36916199

